# The Landscape and Perspectives of the Human Gut Metaproteomics

**DOI:** 10.1016/j.mcpro.2024.100763

**Published:** 2024-04-10

**Authors:** Zhongzhi Sun, Zhibin Ning, Daniel Figeys

**Affiliations:** 1 School of Pharmaceutical Sciences, Faculty of Medicine, University of Ottawa, Ottawa, Ontario, Canada; 2 Department of Biochemistry, Microbiology and Immunology, Faculty of Medicine, University of Ottawa, Ottawa, Ontario, Canada

**Keywords:** metaproteomics, human gut, microbiome, proteome coverage, functional annotation

## Abstract

The human gut microbiome is closely associated with human health and diseases. Metaproteomics has emerged as a valuable tool for studying the functionality of the gut microbiome by analyzing the entire proteins present in microbial communities. Recent advancements in liquid chromatography and tandem mass spectrometry (LC-MS/MS) techniques have expanded the detection range of metaproteomics. However, the overall coverage of the proteome in metaproteomics is still limited. While metagenomics studies have revealed substantial microbial diversity and functional potential of the human gut microbiome, few studies have summarized and studied the human gut microbiome landscape revealed with metaproteomics. In this article, we present the current landscape of human gut metaproteomics studies by re-analyzing the identification results from 15 published studies. We quantified the limited proteome coverage in metaproteomics and revealed a high proportion of annotation coverage of metaproteomics-identified proteins. We conducted a preliminary comparison between the metaproteomics view and the metagenomics view of the human gut microbiome, identifying key areas of consistency and divergence. Based on the current landscape of human gut metaproteomics, we discuss the feasibility of using metaproteomics to study functionally unknown proteins and propose a whole workflow peptide-centric analysis. Additionally, we suggest enhancing metaproteomics analysis by refining taxonomic classification and calculating confidence scores, as well as developing tools for analyzing the interaction between taxonomy and function.

## Highlights

• Evaluate the proteome and protein annotation coverage in human gut metaproteomics.
• Compare the taxonomy of metaproteomics datasets with the metagenomics database.
• Advocate the use of metaproteomics for studying functionally unknown proteins.
• Assess the feasibility of applying peptide-centric analysis in metaproteomics.
• Propose refining the taxon scope and conducting taxon-function cross-analysis.

## Graphical Abstract

**Figure:**
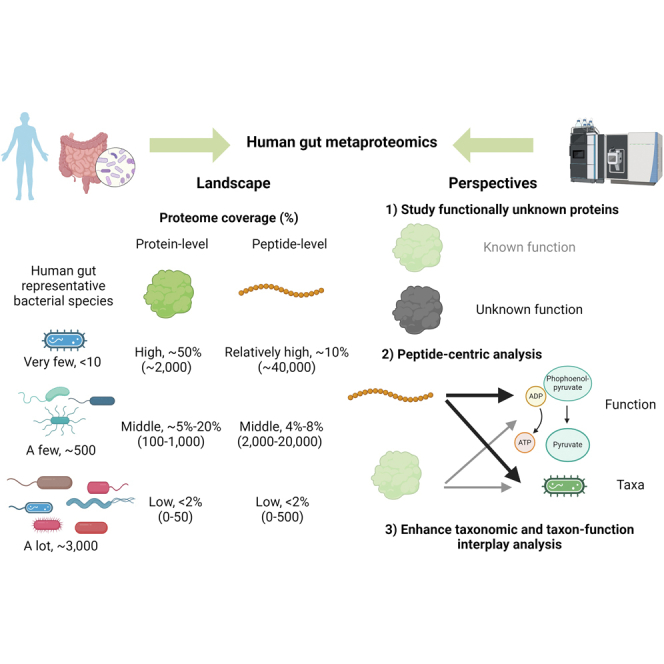

## In Brief

Metaproteomics emerges as a valuable tool for studying the human gut microbiome. This article explores proteome and protein annotation coverage in published human gut metaproteomics datasets. It emphasizes the importance of improving proteome coverage and enhancing functional annotation. The analysis advocates the adoption of peptide-centric analysis and underscores the necessity to improve the integration of taxonomic and functional data in metaproteomic research.

### Applying Metaproteomics to Study the Human Gut Microbiome

The human gut microbiome has complex interactions with the host, and the taxonomic composition and functional activity of the gut microbiome have been closely associated with human health and diseases (1). Proteins are the foundations of most biological processes, comprising about 50% of the dry mass of a cell across different species (2). Their quantification is instrumental in assessing the biomass contributions of different bacterial species within a community (3). Consequently, a comprehensive analysis of the entire protein complement in the microbiome is critical for unraveling host-microbiome interactions.

Metaproteomics was first proposed by Wilmes and Bond in 2004, defined as the “Large-scale characterization of the entire protein complement of environmental microbiota at a given point in time” (4). Initially, metaproteomics involved separating proteins on a 2D gel and manually selecting individual protein spots for mass spectrometric analyses, which was experimentally demanding and low throughput (5). During this early stage of metaproteomics, approximately 2000 proteins could be detected in a microbial community (6). Although it is still in its early stages, the liquid chromatography and tandem mass spectrometry (LC-MS/MS)-based bottom-up metaproteomics is now able to detect approximately 50,000 ∼ 70,000 protein groups in a single study using different techniques (7, 8), showcasing the rapid technological advancement over the past 2 decades.

### The Advantage of Metaproteomics Compared to Other Omics

Metaproteomics measures the presence and abundance of proteins, thereby revealing gene expression dynamics within microbial communities (9). Additionally, by assigning proteins to individual species or higher taxa, metaproteomics offers insights into the taxonomic composition of the microbiota (9, 10). However, metaproteomics encompasses much more than measuring gene expression and species biomass within microbial communities (10). When compared with other high-throughput omics techniques—including amplicon sequencing, metagenomics, metatranscriptomics, and metabolomics—metaproteomics has several unique advantages.

Firstly, metaproteomics is more likely to reveal the real functionality of the microbial community. This contrasts with DNA-based metagenomics and metatranscriptomics, which only suggest the potential functional capabilities of microbial communities. The reason for this disparity lies in the fact that not all DNA (genes) are transcribed into RNA, and not all RNA transcripts are subsequently translated into proteins. Secondly, metaproteomics enables the study of post-translation modifications (PTMs) in microbes. PTMs play a crucial role in modulating protein activity, structural conformation, and interactions, significantly influencing bacterial behavior within the microbiome and in interactions with the host (11). Such modifications, critical for understanding complex biological processes, remain elusive in other high-throughput omics analyses.

In addition, metaproteomics has advantages for studying host-microbe interactions, as both human proteins and microbial proteins are able to be identified and quantified. Human proteins, which constitute approximately 15% of the biomass in metaproteomic samples (12) are pivotal in mediating these interactions. Moreover, metaproteomics can also analyze isotope content, which helps determine carbon sources and provides a deeper understanding of metabolism in microbial communities (13). Overall, metaproteomics distinctively enhances our comprehension of the human gut microbiome, offering insights that are not readily obtainable through other omics technologies.

### Discoveries Achieved Through Metaproteomics

Metaproteomics has emerged as a powerful tool for unraveling the pathogenesis of various diseases and for identifying potential biomarkers. Its application ranges from unraveling the microbial contribution to oxidative stress in inflammatory bowel disease (14) to uncovering the interplay between gut microbiota and the development of type 1 diabetes (15). Metaproteomics has also been applied to exploring host-microbiome interactions underlying other diseases, such as cancer (16, 17), obesity (18, 19), and COVID-19 (20, 21). A comprehensive overview of the clinical applications of metaproteomics has been provided by Wolf *et al.* (22).

Despite these strides, metaproteomics confronts ongoing challenges. While numerous reviews have offered comprehensive insights into research methods (9, 23), accomplishments (22, 24), challenges (25, 26), and future directions (12, 27), few studies offer guidance for advancing metaproteomics based on data from previous studies. Although the protein landscape of human gut bacterial species identified in metaproteomics has been preliminarily explored (28), certain important areas, such as proteome coverage demand further exploration.

In this article, we systematically reanalyzed the identified peptides and proteins from 15 human gut metaproteomics studies compiled in the MetaPep (29) database. Notably, all raw files in our dataset utilized HCD-FTMS for MS2 scans, offering superior resolution and accuracy. All the peptide and protein identification results were acquired from MetaLab-MAG (30), a user-friendly publicly accessible metaproteomics data analysis platform, ensuring the reproducibility of our findings and paving the way for integrating additional datasets in subsequent research.

## Current Landscape of Human Gut Metaproteomics

### Proteome Coverage in Metaproteomics

In the metaproteomics community, it is widely acknowledged that there is still room for enhancing the depth of proteome coverage (31, 32, 33). Although ultra-deep proteomics methods have shown promise in detecting nearly the entire proteome of single-species bacteria, identifying up to 75 to 77% of open-reading frames (34, 35), the issue of limited proteome coverage becomes more significant when dealing with complex microbial communities in metaproteomics. Previous research indicated that increased species diversity reduced the number of identified protein groups, and with the current state of metaproteomics technology, the estimated species and proteome coverage in a complex sample containing around 300 bacterial species is about 20% and 5%, respectively (31). From a more intuitive standpoint, the current stage ultra-deep metaproteomics techniques detected an average of 69,280 peptides, and 30,686 protein groups per microbiome sample (7), these numbers represent a mere 2.34% of the theoretical microbiome proteome, based on an estimated 1,310,000 coding sequences (CDS) (12). These studies suggest that a significant portion of proteins and species remain undetected in metaproteomic analyses, aligning with our previous observations that bacteria with less than 0.5% biomass are difficult to detect using current metaproteomics workflows (36).

While full quantification of all proteins is not necessary to study responses in microbiome networks (37), achieving more comprehensive proteome coverage remains crucial, given its current limitations. To date, total coverage of metaproteomics across human gut bacterial species and functions has not been thoroughly investigated. Recently, MetaPep (29) compiled identified peptides from over 2000 human gut metaproteomics raw files from 15 published studies, allowing us to comprehensively evaluate the extent of proteome coverage and the scope of detectable proteins within the metaproteomics field. These peptides were identified by searching raw files from each study with MetaLab-MAG. During the search, MetaLab-MAG integrated a human proteome fasta file from UniProt into the search space. Carbamidomethyl[C] was set as a fixed modification, with Oxidation[M] and Acetyl[ProteinN-term] as variable modifications. All other parameters were kept at their default settings. To refine our focus on microbial peptides, peptides exclusively found in the human proteome were excluded. Out of the compiled 1,163,940 peptides in MetaPep, only 2837 were found in both bacterial and human proteomes.

#### Peptide-Level Proteome Coverage

We first directly investigate the proteome coverage at the peptide level. To be specific, peptides identified in human gut metaproteomic studies were mapped to the species or lowest common ancestor (LCA) of human gut bacteria from the UHGG (Unified Human Gastrointestinal Genome) dataset as described in the original MetaPep publication (29). These peptides were further mapped to the phylogenetic tree of representative bacterial species from the UHGG dataset (38) and visualized with iTOL (39). Our analysis revealed several interesting patterns.

**Fig. 1:**
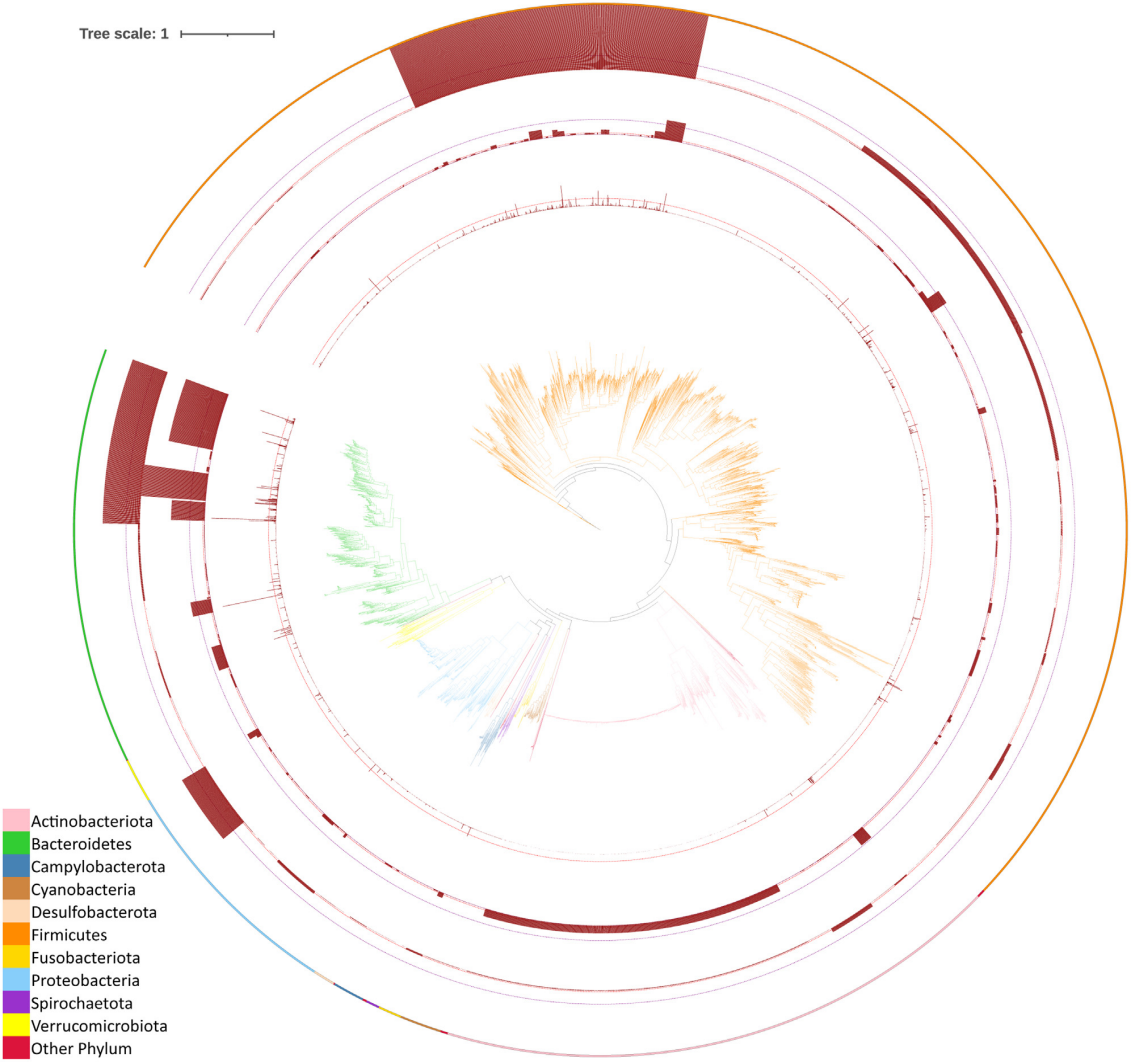
**Phylogenetic distribution of identified peptides in metaproteomics studies.** The innermost is the phylogenetic tree of 4716 bacterial species extracted from the UHGG dataset. From the inner to the outer circle, 3 bar plots show the number of genome-distinct peptides, peptides assigned to genus-level lowest common ancestors (LCA), and peptides assigned to family-level LCA for each node/clade. Dashed lines serve as references for the number of peptides: *red dash line* (1000 peptides), *purple dash line* (10,000 peptides). Phylum-level taxonomy is indicated by *different colors* in both the phylogenetic tree and the color strip on the outermost ring.

First, we noted an uneven phylogenetic distribution of identified peptides across human gut bacteria (Fig. 1). Out of the total 1,163,940 peptides from MetaPep, 293,181 (25.2%) could be assigned to 4110 of the 4744 representative prokaryotic species (4716 bacterial and 28 archaeal) in the UHGG database. These peptides that were found exclusively in one prokaryotic species are referred to as genome-distinct peptides. In contrast to the high proportion (84.5%) of genome-distinct peptides among all in-silico digested peptides of UHGG representative genomes (29), the ratio of genome-distinct peptides in MetaPep is much lower. This difference is reasonable because genome-distinct peptides, unlike peptides shared by multiple species, are expected to have a lower abundance in real microbial samples. This lower abundance makes it more challenging to detect genome-distinct peptides, resulting in fewer of them being collected in MetaPep. Focusing on the analysis of MetaPep compiled peptides, the number of genome-distinct peptides of each bacterial species varied from 0 to 9093. Peptides assigned to higher taxonomic levels, such as genus and family, were also incorporated into the phylogenetic mapping (Fig. 1). For peptides assigned to genus level LCA, five genera (*Bacteroides*, *Prevotella*, *Phocaeicola*, *Parabacteroides*, *Blautia_A*, and *Faecalibacterium*) have >10,000 peptides. For peptides assigned to family level LCA, three families (Lachnospiraceae, Bacteroidaceae, and Enterobacteriaceae) have >10,000 peptides. Species from these taxonomic units also had a larger number of genome-specific peptides (Fig. 1), indicating these bacteria were frequently identified from human gut metaproteomics. While it is essential to consider these taxonomic units with numerous identified peptides, it is equally important to note that many bacterial species (2198) were represented by only a limited number of genome-distinct peptides (≤5), including 646 species that had no identifiable genome-distinct peptides. These 2198 species accounted for 46.6% of the 4716 representative bacterial species in the UHGG database. At the family level, the most represented sources of these species were Coriobacteriaceae (566), Acutalibacteraceae (104), Oscillospiraceae (99), UBA660 (91), and CAG-508 (40). At the genus level, the most represented were *Collinsella* (564), *Streptococcus* (41), *Veillonella* (28), *CAG-1427* (25), and *CAG-269* (23). There was no overlap between the taxa with the highest number of identified peptides and those with the most species lacking identifiable peptides, highlighting the uneven phylogenetic distribution of identified peptides across species and indicating that some bacterial taxa are rarely detected in the compiled metaproteomics datasets.

Second, we observed that the number of identified peptides for each bacterial species in metaproteomics constitutes only a small fraction of that species' entire proteome. *Phocaeicola dorei*, for example, has the highest number of genome-distinct peptides (9093) in MetaPep, with 60 additional species also having more than 1000 genome-distinct peptides each (Fig. 1). Focusing on *P. dorei*, 38,293 peptides from MetaPep could be assigned to this bacteria species, as more peptides assigned to higher taxonomic levels could also be found in this species. However, these 38,293 peptides only account for 9.71% of all 394,374 *in silico*-digested peptides of this species (in-silico digestion parameters: minimum peptide length = 7, maximum missed cleavage = 2). For the other 60 species with over 1000 genome-distinct peptides, an average of 12,748 peptides from MetaPep corresponded to 1.61 to 9.58% of each species' in-silico digested peptides. This indicates that even for species with the most identified peptides, only a small proportion of their theoretical proteome is detected. The number of identified peptides for all bacterial species is shown in Supplemental Table S1.

#### Protein-Level Proteome Coverage

We also investigated proteome coverage at the protein level by re-examining protein identification results from metaproteomics studies collected in MetaPep (29). In total, 306,413 unique head proteins were extracted from all identified protein groups across 15 studies. The head protein, which is the first protein listed in a protein group identified by MetaLab-MAG (30), shares identified peptides with all other proteins in the group. The overall landscape of metaproteomics studies, as revealed by analyzing these head proteins, was similar to the findings at the peptide level.

**Fig. 2:**
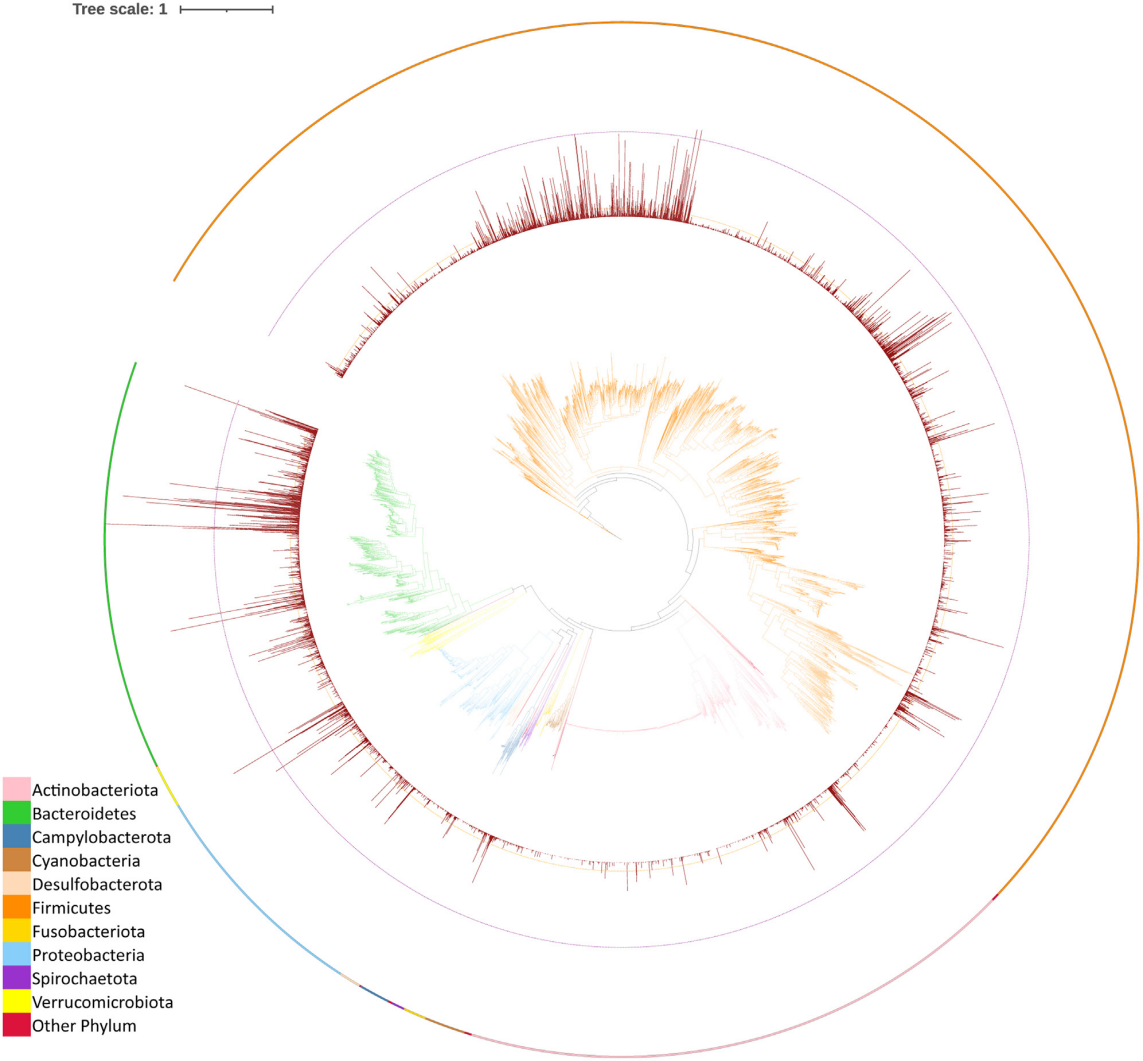
**Phylogenetic distribution of identified proteins in metaproteomics studies.** The innermost is the phylogenetic tree of 4716 bacterial species extracted from the UHGG dataset. The bar plot shows the number of identified proteins from each bacterial species. *Dashed lines* serve as references for the number of proteins: *red dash line* (100 proteins), *purple dash line* (1000 proteins). Phylum-level taxonomy is indicated by *different colors* in both the phylogenetic tree and the color strip on the outermost ring.

First, similar to the peptide-level analysis, an uneven phylogenetic distribution was observed among the identified proteins. The head proteins from identified protein groups originated from 4288 of the 4744 representative prokaryotic species in the UHGG, and the number of identified proteins per species varied widely, from 0 to 2282 (Fig. 2). Consistent with peptide-level results, in protein-level analysis, the same five genera (*Bacteroides*, *Prevotella*, *Phocaeicola*, *Parabacteroides*, *Blautia_A*) had the most identified head proteins, exceeding 10,000 each. At the family level, the same three families (Lachnospiraceae, Bacteroidaceae, Ruminococcaceae) had the most identified head proteins, with two other families Enterobacteriaceae and Oscillospiraceae, also having more than 10,000 identified head proteins. In parallel with taxa with numerous identified proteins, a significant number of bacterial species and taxa were represented by only a limited number of identified proteins (Fig. 2).

Second, the number of identified proteins covered a limited portion of the proteome of bacterial species. Similar to the peptide-level coverage results, at the protein level, *P. dorei* also has the largest number of identified head proteins (2282), covering 50.5% of the species proteome (all 4522 CDS). However, the majority of species (3655) exhibited limited proteome coverage (<5%), with fewer species showing higher coverage (199 genomes with 10 to 20% coverage, 83 genomes with 20 to 50% coverage, and only two genomes with ≥50% coverage). The number of identified head proteins of each bacterial species is also shown in Supplemental Table S1. While it is hard to estimate how many in-silico digested peptides are present in the sample (40), it is well-established that the majority of bacterial coding sequences are indeed expressed as proteins (41, 42). Compared to peptide-level analysis, analyzing at the protein level provides a more intuitive understanding of proteome coverage.

### Comparison of Taxonomic Composition Between Bacterial Species Identified in Metaproteomics Studies and the Species in the Metagenomics Reference Database

**Fig. 3:**
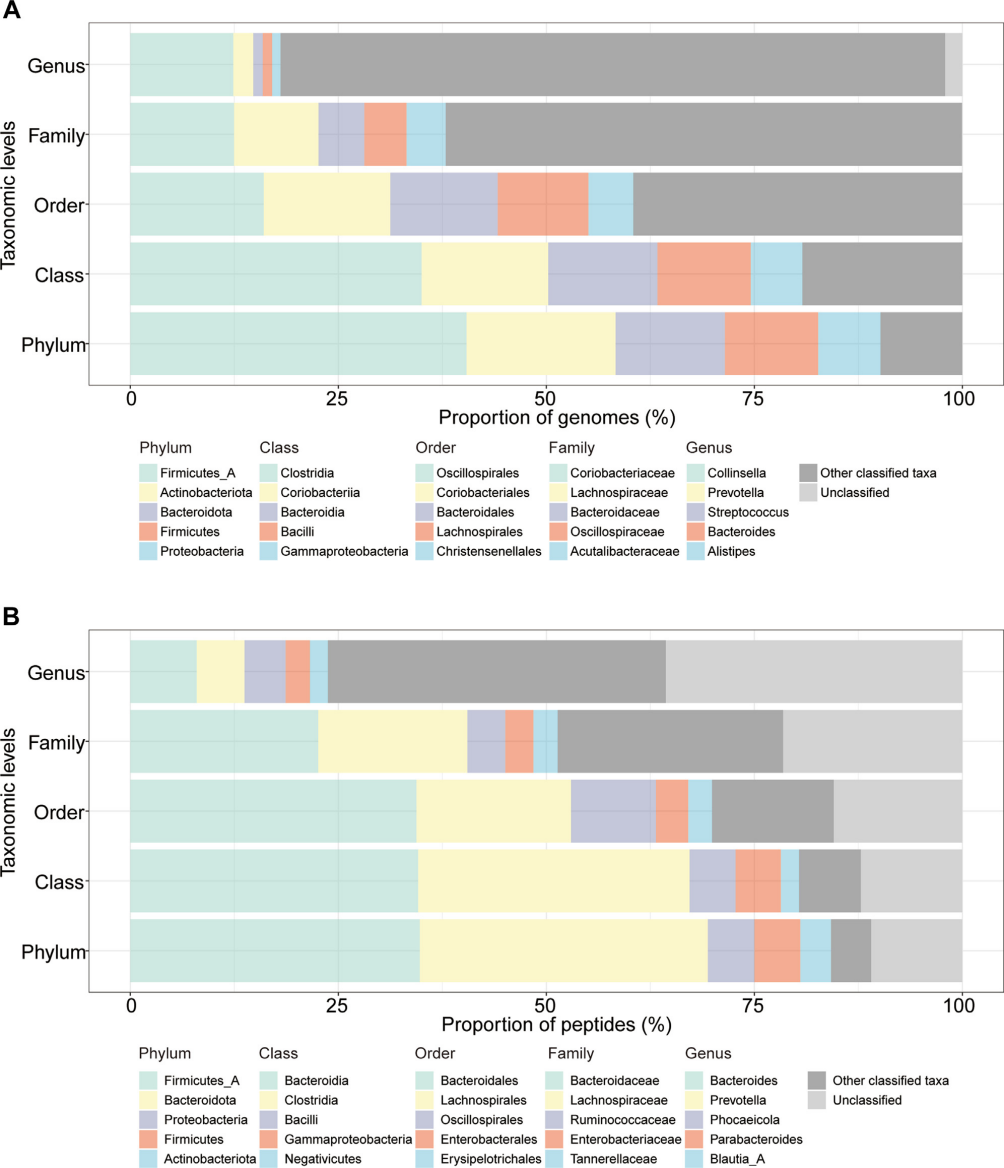
**Comparison of taxonomic composition of bacterial species identified in metaproteomic studies and the species in the metagenomics reference database, UHGG.***A*, taxonomic affiliation of the bacterial species collected in the UHGG at different taxonomic ranks. *B*, taxonomic affiliation of the metaproteomics-identified peptides (peptides compiled in MetaPep) at different taxonomic ranks. The legend only depicts the five most highly represented taxa per rank.

In addition to only focusing on the metaproteomics dataset, we further compared the taxonomic composition of bacterial species identified in metaproteomics studies and the species in the metagenomics reference database. We extracted the taxonomic composition at various taxonomic ranks from the metagenomics reference database, UHGG (38), as presented in Figure 3*A*. On the other hand, the number of identified peptides collected in the MetaPep and their taxonomic sources at different taxonomic ranks are shown in Figure 3*B*.

At higher taxonomic ranks, the overarching taxonomic composition of the bacterial species identified in metaproteomic analysis aligns with the composition of representative genomes derived from the UHGG (Fig. 3, *A* and *B*). At the phylum level, the predominant groups—Firmicutes_A, Bacteroidota, Proteobacteria, Firmicutes, and Actinobacteriota—were not only the most genome-rich in the UHGG but also yielded the highest number of identified peptides in MetaPep. However, their relative abundances vary between the metagenomic reference database and metaproteomic datasets. For instance, Bacteroidota comprises approximately 15% of the UHGG's genomes but contributes around 34.6% of MetaPep's peptides. Conversely, Actinobacteriota accounts for about 18% of UHGG genomes, but only3.7% of peptides in MetaPep. The top taxa at the class and order levels were generally consistent between the two datasets, with minor exceptions (Fig. 3, *A* and *B*).

At the family and genus levels, however, this congruity wanes, with only two taxa shared across the five most represented taxa in the metagenomics reference database and metaproteomics datasets (Family level: Bacteroidaceae and Lachnospiraceae; Genus level: *Bacteroides* and *Prevotella*). Notable discrepancies include *Collinsella*, which, despite possessing the highest number of genomes at the genus level in the UHGG database (584, representing 12.3%), accounts for a mere 0.6% of peptides (5745) in MetaPep. Similarly, *Streptococcus*, while being the third most genome-abundant genus (54, or 1.1% of UHGG genomes), corresponds to only 0.3% of MetaPep's peptides. In contrast, *Parabacteroides*, with only 27 genomes in the UHGG, constitutes 2.9% of the peptides (27,279) in MetaPep. The differences in the taxonomic composition at the family and genus levels were also observed when considering more representative taxa units. Among the 30 families and 30 genera with the highest number of identified peptides in metaproteomics studies, 17 families and 13 genera overlapped with taxa units that had the highest number of genomes in the UHGG.

In summary, while there are consistent representations of higher taxonomic ranks (phylum, class, and order) across both the metagenomic reference database and metaproteomic datasets, substantiating their abundance in the human gut microbiome, discrepancies at lower taxonomic ranks highlight the unique insights afforded by metaproteomic analysis. These differences underscore the value of metaproteomics in providing a complementary angle for analyzing the structure of microbial communities by assessing species biomass (3).

### Metaproteomics View of Unannotated Proteins

#### Importance of Protein Functional Annotation

Protein functional annotation is fundamental for the understanding of biological processes, unfortunately, many genes from the microbiome are not or are poorly annotated and we need new approaches to address this challenge. The advent of sequencing technologies has resulted in the discovery of a plethora of new gene sequences, while, the experimental characterization of their protein products and function remains unaddressed, leading to a widening sequence-to-function gap (43). Even for well-studied organisms like *Homo sapiens* and *Escherichia coli*, 10% (44) and 7.2% (45) of proteins, respectively, remain unannotated. The challenge is more pronounced in the complex human gut microbiota, where many species lack cultured representatives, and a larger fraction of microbial proteins lack functional annotation. The UHGG database highlights this issue, revealing that 27.3% of genes do not match any functional database, and an additional 14.2% of genes match COG (Cluster of Orthologous Groups) categories with unknown functions (38), resulting in a total of 41.5% of genes poorly annotated. It is likely that considering ∼50,000 metabolites potentially produced by the gut microbiome (46), some proteins are moonlighting which might not be captured in their annotation (47, 48).

#### Impact of Unannotated Proteins on Metaproteomics

The unannotated proteins have two significant impacts on metaproteomics studies. The first impact is that most metaproteomics analyses rely on protein database searches for peptide and protein identification. However, existing prokaryotic gene prediction tools accurately detect only 60 to 70% of start codons for specific bacterial species (49), leading to potential misannotations or omissions. This directly impacts the results of sequence database searches. The second impact is on the interpretation of metaproteomics data. Similar to other high-throughput omics studies, most metaproteomics studies start with pathway or network analysis to identify biological processes that significantly changed, where functional annotations of proteins are crucial. Annotation biases can result in a skewed understanding, focusing on a limited set of annotated proteins while overlooking the functions of many others (50). Indeed, some unannotated proteins have been implicated in disease development and may have essential roles (51, 52).

#### Unannotated Proteins in Genomics and Metaproteomics Datasets

Annotation coverage of different bacterial species has been systematically investigated in genomics data, with an average of 52 to 79% of the coding sequences in bacterial genomes could be functionally annotated, and the annotation coverage ranging from 14% in some species to 98% in others (53). This disparity suggests that taxonomy is a major factor influencing annotation completeness. However, the annotation coverage of proteins identified in metaproteomics studies has not been systemically investigated. Here, we revisited the annotations of 306,413 identified head proteins, sourced from studies collected in MetaPep (29). We discovered that 78,474 (25.6%) of these proteins have neither KEGG ko (54) nor Gene Ontology (GO) annotation (55, 56). Among these proteins, 28,100 (9.17%) proteins either had no COG category annotation or were assigned to COG category S (Function unknown), indicating limited functional information available for these proteins.

Interestingly, a higher proportion of proteins identified in metaproteomics have functional annotations compared to the overall gene datasets in UHGG. While 41.5% of genes in UHGG are poorly annotated, only 9.17% of metaproteomics-identified proteins lack annotations. Notably, among the 28,100 unannotated metaproteomics proteins, 2342 were highly abundant (within the top 10% of total intensity in specific studies), suggesting that these functionally unknown proteins could play significant biological roles. This underlines the potential of metaproteomics in investigating the functions of these enigmatic proteins.

When we analyze the ratio of annotated proteins among those identified from each bacterial species, we observe that the majority of identified proteins from different species already have functional annotations (see Supplemental Table S1). For species with at least 100 identified proteins, the percentage of proteins with annotations ranges from 79.0 to 100% of all the identified proteins. Notably, *Bacteroides* sp900765785 and *Bacteroides fragilis* have the lowest proportions of annotated proteins among the identified proteins, at 79.0% and 79.9%, respectively.

### Peptide-Centric Analysis

#### Challenges of Protein Inference and the Advantages of Peptide-Centric Analysis

Protein inference poses another challenge in metaproteomics. Within a complex microbial community, a single peptide could be shared by hundreds of different proteins, complicating the precise attribution of the peptide to its parent protein. As a result, in metaproteomics, protein inference usually does not yield a list of proteins, but rather a list of protein groups. These protein groups can encompass proteins from various bacterial species with different functions, resulting in the loss of valuable information during protein inference (57). Moreover, different protein inference algorithms can produce protein group lists with substantial differences (58, 59). All these factors in protein inference further impact subsequent taxonomic and functional analysis.

Since mass spectrometry intrinsically identifies peptides, not proteins, a peptide-centric approach for functional and taxonomic analysis emerges as a logical alternative in metaproteomics. Tools such as Unipept (60, 61), PepFunk (62), and MetaGOmics (63) facilitate this approach, linking peptides directly to their functional and taxonomic attributes. This method bypasses the protein inference step, building the microbial community profile based on peptide identifications and quantifications. Research indicates that peptide-centric analysis can offer enhanced sensitivity and uncover details that protein-level analysis might miss (64, 65). Moreover, our recent work on MetaPep (29) also substantiated the feasibility of initiating peptide-centric analysis by searching a peptide sequence database. Notably, this database is significantly smaller than its protein counterpart, offering a considerable advantage in terms of reducing search times throughout the peptide-centric analysis workflow.

#### Feasibility of Peptide-Centric Analysis

The feasibility of peptide-centric taxonomic analysis was demonstrated with the introduction of the first tryptic peptide-based metaproteomics biodiversity analysis method, Unipept (60), in 2012. Since then, peptide-centric taxonomic analysis has gained widespread adoption in metaproteomics (15, 16, 66), with subsequent developments in Unipept enabling functional analysis *via* GO terms and EC numbers (61). However, concerns have been raised about the limited sequence length of peptides and their capacity to convey meaningful functional information (67, 68). A proposed solution involves tailoring the protein sequence collection for in-silico digestion, ensuring that each peptide correlates to a protein with a specific function. Customized or research-specific databases for peptide digestion have been shown to enhance functional resolution in peptide-centric metaproteomics analyses (69).

To verify the feasibility of peptide-centric function analysis for human gut metaproteomics studies, we performed a preliminary test on the UHGG database for annotating in-silico digested peptides from the database to specific functions. Out of the 10,234,935 proteins from 4744 representative prokaryotic species within the UHGG database, 8,277,932 (80.9%) of them had specific COG family functional annotations. We collected proteins from each COG family, along with proteins lacking COG annotations, for in-silico digestion. This process yielded a total of 392,459,520 peptides, with 385,535,220 being unique peptide sequences. After digestion, 325,260,860 (84.3%) of these unique peptides were exclusively found in proteins from specific COG families and 55,404,896 (14.4%) were exclusive to proteins without COG family annotations. And these peptides were referred as to functional-distinct peptides. The remaining 4,869,464 unique peptides were shared among proteins from multiple COG families, accounting for only 1.3% of the total unique peptides. These findings suggest that a substantial majority of identifiable peptides can be confidently associated with specific functional categories.

## Perspectives of the Human Gut Metaproteomics

### Improving the Coverage of Human Gut Metaproteomics

#### Scaling up Metaproteomic Studies

The limitation in metaproteomics coverage was not only confined to individual studies but was also reflected in the overall limited number of metaproteomics studies. Despite an uptick in the quantity of metaproteomic research, there remains a stark contrast when compared to metagenomics. According to the search results from the *Web of Science*, during the past 10 years (2013–2022), 12,298 papers with topics in metagenomics have been published, while only 770 papers with topics in metaproteomics were published.

The human gut microbiome research has seen substantial expansion in gene catalogs such as IGC (70), UHGG (38), UNITN (71), and so on. In contrast, there is a paucity of efforts directed toward curating and reanalyzing peptides and proteins from available metaproteomic datasets in repositories like PRIDE (72), and ProteomeXchange (73). While Stamoulian *et al.*, examined over a thousand metaproteomics raw files to identify generalist species expressed across all samples and specialist species that are highly expressed in a small subset of samples associated with a certain phenotype (28), their article does not focus on the proteome coverage of each microbial species. Conversely, our recently developed MetaPep (29) dataset, which compiled identification results from over 2000 raw files across 15 published metaproteomics studies, may serve as a robust resource to enhance our understanding of metaproteomic coverage. The expectation is that the assembly of more extensive peptide and protein datasets will further enrich our knowledge of the human gut metaproteome.

#### Advancing Coverage with DIA and Emerging Technologies

Instrumental limitations notably affect metaproteomic coverage. Traditional mass spectrometry's capability to acquire tandem mass spectra within a given time frame is inherently limited. Even given enough scanning time, not all digested peptides in the sample are detected by mass spectrometry. MS-based proteomics tends to identify proteotypic peptides (38). For instance, in conventional data-dependent acquisition (DDA) methods typically, less than 1% of incoming precursor ions are fragmented and identified by MS2 (74).

Data-independent acquisition (DIA) metaproteomics, has shown improvements in proteome coverage, reproducibility, and accuracy in quantification over DDA methods (75). Instead of acquiring MS/MS scans with narrow isolation windows centered on peptide precursors detected in an MS scan in the traditional DDA, DIA acquires MS/MS scans with wide isolation windows that do not target any particular precursor (76). Additionally, the cutting-edge DIA-PASEF (Parallel Accumulation-Serial Fragmentation) is in theory sampling up to 100% of the peptide precursor ion (74). The application of DIA-PASEF in mouse microbiome studies, which potentially doubled protein identifications, underscores its promise (77). However, the application of such advanced methodologies to human gut microbiome studies is in its infancy, hindered by the nascent state of requisite bioinformatics pipelines and the complexity inherent in the technique.

However, it should be noted that DIA-based metaproteomics is only going to make a small dent in the dark field of the metaproteome. Novel enrichment techniques specifically designed for the human gut microbiome will likely be necessary to obtain a larger coverage of the metaproteome. For instance, the application of activity-based probes (ABPs) has facilitated the enrichment of proteins possessing distinct functionalities, thereby enabling the identification and quantification of proteins that are present at levels below conventional detection thresholds (78). Furthermore, various enrichment strategies also have substantially elevated the number of small proteins that are typically challenging to detect through standard metaproteomic methodologies (79).

### Enhancing Protein Annotation Through Metaproteomics

Biochemical and genetic experiments are traditional methods for elucidating protein functions. In the absence of experimental data, proteins that have not been characterized are often assumed to have the same functions as proteins that have been experimentally characterized and share high sequence similarity. Surpassing traditional alignment-based techniques, recent advances have introduced machine learning and deep learning approaches to bolster protein function annotation (80, 81). Additionally, protein structure families based on clustering the predicted structure of nearly every known protein have expanded the dimensions of the protein universe, revealing that most protein structures are not functionally dark, shedding light on the functional annotation of a broader array of proteins (82, 83).

Proteomics data has been suggested as a valuable resource for enhancing protein functional annotation (37, 84). A study has cataloged the proteome-wide protein abundance of *E. coli* in response to more than 100 genetic perturbations, casting light on the modulation of functionally linked proteins and providing mechanistic insights into this model organism (85). Similarly, metaproteomics data can also contribute to protein function annotation by identifying proteins with consistent changes in abundance under different treatments. These changes indicate similarities or connections in their roles. However, in the analysis of metaproteomics samples, it is important to consider the abundance changes of different taxa. Given the presence of numerous functionally unknown proteins with high abundances in metaproteomics samples, mining metaproteomics data holds the potential to uncover the functions of these mysterious entities.

### Performing Peptide-Centric Analysis in Metaproteomics

Our findings in this article demonstrate that confining the search space to meticulously curated such as the UHGG representative prokaryotic species, enables the comprehensive annotation of most peptides with detailed functional and taxonomic information. This outcome, coupled with the demonstrated utility of peptide-centric analysis tools like UniPept (61), pepFunk (62), and MetaGOmics (63) reinforces the feasibility of peptide-centric analysis in metaproteomics.

Nevertheless, the current peptide-centric analysis mainly focuses on downstream analysis after the sequence database search, while most metaproteomics studies still search protein sequence databases. Given that the identified peptides only account for a small part of all in-silico digested peptides from proteins (86), searching a protein database increases computational complexity. To achieve a comprehensive peptide-centric analysis workflow and reduce computing resource consumption, a refined and well-annotated peptide sequence database is required. Fortunately, the advent of innovative methodologies for predicting peptide detectability (87, 88) holds promise for condensing the search space, transitioning from an exhaustive protein sequence database to a more concise peptide sequence database. Implementing a complete peptide-centric analysis workflow initiated with searching a peptide database search, is anticipated to expedite metaproteomics data processing and enable more expansive studies in this field.

### Enhancing Taxonomic and Taxon-Function Interplay Analysis

#### Refining Taxon Scope and Calculating Confidence Scores to Improve Taxonomic Analysis

The choice of protein sequence databases is known to significantly impact the outcome of metaproteomics (89). Moreover, the delineation of the search space establishes distinct parameters for analyzing the taxonomic composition of the sample. Employing preliminary amplicon sequencing, metagenomics, or using metaproteomics data alone for predetermining and refining the taxonomic scope could enhance the precision of taxonomic analyses within metaproteomics. However, the current metaproteomics analysis pipelines lack the flexibility to customize the taxonomic scope. The incorporation of this capacity is likely to enhance not just the detail of taxonomic resolution but also the efficiency of metaproteomics searches.

In addition, when applying metaproteomics to study complex microbiome samples with multiple species, inferring the presence of taxa based on identified peptides can be a complex endeavor. While existing metaproteomics analysis platforms such as MetaLab MAG employ Occam's razor for species referencing, the reliability of identified bacterial species remains largely unknown. A confidence score calculation method has been applied for strain-level taxonomic assignment of viral proteome samples (90). Developing meaningful confidence scores represents a promising direction for reinforcing the metaproteomic toolkit's ability to dissect microbial community structures.

#### Advancing Tool Development for Taxon-Function Crosstalk Analysis

In the realm of omics analyses, simply compiling a list of microbial taxa and gene/protein abundances is increasingly recognized as insufficient. Going beyond this, the identification of microbes and their corresponding functional contributions to the microbial community provides novel insights (91, 92). While tools such as BURRITO (93) and MetaFunc (94), have bridged taxonomic and functional data, the broader scientific community still faces challenges when attempting to conduct taxon-function crosstalk analysis. Such analyses are invaluable as peptides/proteins inherently embody both taxonomic and functional data. Therefore, the development of analytical tools and methodologies aimed at linking taxonomic identity with functional activity is a vital direction for advancing metaproteomic research.

## Summary

This paper presents a comprehensive analysis of the proteome coverage of human gut bacterial species identified in metaproteomics studies. To address the challenge of limited proteome coverage, there is a growing need for expanded metaproteomic research and the advancement of innovative methodologies. We also highlight the high annotation coverage of proteins identified through metaproteomics, indicating the significant potential of metaproteomics in improving protein annotation. Additionally, we demonstrate the feasibility of peptide-centric analysis as a promising approach to reduce computational demands in metaproteomics data analysis. The paper discusses various perspectives on enhancing the reliability of taxonomic analysis and facilitating taxon-function interactions in metaproteomics. These combined efforts aim to leverage metaproteomics in enhancing our understanding of microbial ecosystems and their complex interactions with host systems.

## Data Availability

Additional data are available upon request.

## Supplemental data

This article contains supplemental data.

## Conflict of interest

D. F. is a co-founder of Biotagenics and MedBiome, both of which are clinical microbiomics companies. The other authors declare no competing interests.
